# *In Silico* Identification of New Targets for Diagnosis, Vaccine, and Drug Candidates against *Trypanosoma cruzi*

**DOI:** 10.1155/2020/9130719

**Published:** 2020-12-10

**Authors:** Rafael Obata Trevisan, Malú Mateus Santos, Chamberttan Souza Desidério, Leandro Gomes Alves, Thiago de Jesus Sousa, Letícia de Castro Oliveira, Arun Kumar Jaiswal, Sandeep Tiwari, Weslley Guimarães Bovi, Mariana de Oliveira-Silva, Juliana Cristina Costa-Madeira, Lúcio Roberto Cançado Castellano, Marcos Vinicius Silva, Vasco Azevedo, Virmondes Rodrigues Junior, Carlo José Freire Oliveira, Siomar de Castro Soares

**Affiliations:** ^1^Department of Microbiology, Immunology and Parasitology, Federal University of Triângulo Mineiro, Uberaba, Minas Gerais, Brazil; ^2^Department of Genetics, Ecology and Evolution, Federal University of Minas Gerais, Belo Horizonte, Minas Gerais, Brazil; ^3^Human Immunology Research and Education Group-GEPIH, Technical School of Health, Federal University of Paraíba, João Pessoa, Paraíba, Brazil

## Abstract

Chagas disease is a neglected tropical disease caused by the parasite *Trypanosoma cruzi*. Despite the efforts and distinct methodologies, the search of antigens for diagnosis, vaccine, and drug targets for the disease is still needed. The present study is aimed at identifying possible antigens that could be used for diagnosis, vaccine, and drugs targets against *T. cruzi* using reverse vaccinology and molecular docking. The genomes of 28 *T. cruzi* strains available in GenBank (NCBI) were used to obtain the genomic core. Then, subtractive genomics was carried out to identify nonhomologous genes to the host in the core. A total of 2630 conserved proteins in 28 strains of *T. cruzi* were predicted using OrthoFinder and Diamond software, in which 515 showed no homology to the human host. These proteins were evaluated for their subcellular localization, from which 214 are cytoplasmic and 117 are secreted or present in the plasma membrane. To identify the antigens for diagnosis and vaccine targets, we used the VaxiJen software, and 14 nonhomologous proteins were selected showing high binding efficiency with MHC I and MHC II with potential for *in vitro* and *in vivo* tests. When these 14 nonhomologous molecules were compared against other trypanosomatids, it was found that the retrotransposon hot spot (RHS) protein is specific only for *T. cruzi* parasite suggesting that it could be used for Chagas diagnosis. Such 14 proteins were analyzed using the IEDB software to predict their epitopes in both B and T lymphocytes. Furthermore, molecular docking analysis was performed using the software MHOLline. As a result, we identified 6 possible *T. cruzi* drug targets that could interact with 4 compounds already known as antiparasitic activities. These 14 protein targets, along with 6 potential drug candidates, can be further validated in future studies, *in vivo*, regarding Chagas disease.

## 1. Introduction

Chagas disease is a neglected tropical disease that affects around 8 million people worldwide, caused by the protozoan *Trypanosoma cruzi* [1]. It is primarily transmitted by blood-borne vectors such as triatomines and also through oral ingestion, blood donation, and organ transplantation, especially in countries where blood donors are not screened for *T. cruzi*. Clinical manifestations of the disease may vary from severe myocarditis and/or changes in the gastrointestinal system, presenting megaesophagus and/or megacolon, to an asymptomatic or undetermined form [2]. During these phases, individuals have a strong cellular and humoral immune response [3, 4]. Despite these cellular and humoral immune responses, there is no diagnostic antigen yet to characterize and identify each of these phases of the disease. Still, it is not clear which mechanisms trigger the transition from asymptomatic to symptomatic but what is known are the factors involved in the etiopathogenesis that are related to the parasite strains, parasite load, infection phase, and the host immune response [5].

The development of an anti-*T. cruzi* vaccine has proven to be a challenging task due to difficulties of finding antigens or formulations with effective protection and also because of the risk of developing autoimmunity, which is considered by many to be a potential cause of disease progression and/or pathogenesis [6]. In this regard, a wide variety of vaccine formulations have been tested over the past decade, thus providing strong evidence that *T. cruzi* can be controlled by vaccines in experimental models. However, these studies have shown that vaccine formulation and vaccine antigens are still not satisfactory; thus, further studies are essential to obtain a vaccine that is truly effective for the entire population [6, 7].

The vaccines developed using conventional approaches and already tested in experimental models are based on inactivated or attenuated pathogens or isolated parasite antigens. Although successful in several cases, these vaccines were not enough for their approval or multicenter trials in humans [8–10]. Regarding the vaccines based on previously selected antigens, there are still few studies investigating such antigen properties, being necessary to explore their origin and diversity, as well the most important epitopes and immune response activated against each one [6, 7]. The application of computational methods to analyze the immunological process, known as immunoinformatics, is revolutionizing vaccine development. In this regard, reverse vaccinology is approachable to identify vaccine candidates in the postgenomic era with reduced cost and time. It is a genome-based screening of epitopes for B and T cells from predicted proteins that can elicit an immune response. First, conserved proteins among all strains of any species of interest are predicted using immunoinformatics approaches such as pan-genomics aimed at finding common vaccine targets against all pathogen strains. Since vaccines may eventually induce an autoimmune response, it is important to analyze conserved predicted proteins against host proteins through a subtractive genomics approach. The subcellular localization prediction is also needed since the membrane and secreted proteins are the first to contact the host. Then, MHC I and II bindings are predicted, looking forward to possible diagnosis and vaccine targets. All stages are aimed at filtering targets until they reach what is most likely to generate an effective immune response [11].

The available drug used for Chagas disease treatment presents various side effects and variable cure rates at different stages of the disease. Moreover, these drugs were developed in the 1970s when there are only a few studies focused on this area. Considering this, molecular docking analysis is a useful tool that describes the interaction between small molecules (compound/ligand) with active sites, receptor residues (protein) of interest [12]. This approach is considered successful when it can identify the nearest ligand with the receptor, discovering the geometrical shape of the ligand within a boundary of specific obstructions and their connections [12]. It had become an important computational technique, playing an essential role in drug discovery against various pathogens.

Thus, this present study is aimed at identifying potential diagnostic and vaccine candidates and pharmacological targets for *T. cruzi*/Chagas disease using subtractive genomics, reverse vaccinology, and molecular docking tools.

## 2. Material and Methods

### 2.1. Data Collection, Gene Prediction, and Orthology Analyses

The complete genome sequences of 28 *T. cruzi* strains were obtained from the GenBank database, available on the National Center for Biotechnology Information (NCBI). Then, the GeneMark group software was used for gene prediction, homogenizing the predictions in order to avoid unexpected results and possible misinterpretations.

The orthology of the predicted proteins was determined using OrthoFinder through standard mode parameters in the Diamond tool (v0.9.22.123) all-versus-all. OrthoFinder is a fast, accurate, and comprehensive analysis tool used in comparative genomics. This software finds orthologists and orthogroups, determining phylogenetic trees, and also provides comprehensive statistics for comparative genomics analysis [13].

### 2.2. Identification of Intraspecies Conserved Proteins Nonhomologous to the Host

Vaccine and drug targets must avoid autoimmune responses, and diagnostic targets must be molecules specific for only one microorganism. For this, subtractive genomics was carried out to identify, in the core, nonhomologous proteins to the host. We used BLASTp from the core genome against human proteins, which are found in databases provided by NCBI; proteins from the core genome that were homologous to human proteins were excluded.

### 2.3. Protein Subcellular Localization

The proteins were evaluated for their subcellular localization using PSORT [14] and von Heijne signal sequence recognition [15]. PSORT predicts the presence of signal peptide by the McGeoch method [14], which is considered an N-terminally charged region and a central hydrophobic region. A score is calculated from 3 values: length of the hydrophobic region, the peak value of that region, and the net charge in the N-terminally charged region. Thus, a large positive discriminant score indicates a high possibility of having a signal sequence, whether cleaved or not. After PSORT, a second method is used: von Heijne signal sequence recognition [15]. This is a weight matrix method and incorporates information around the cleavage site, meaning it can detect signal sequences or not. A large positive output means more chances of being a cleavable signal sequence [16]. Data generated by PSORT classify proteins as cytoplasmic, secreted (those that present signal to the endoplasmic reticulum and secretory system vesicles), nuclear, or membrane proteins. We submitted to MHOLline the multifasta files containing all amino acid sequences, regardless of their subcellular location [17]. This online tool uses various dependencies such as HMMTOP, BLAST, BATS, MODELLER, and PROCHECK to predict the three-dimensional modeling of target proteins. Only very high, high, good, and medium to good quality sequences were used from MHOLline classified groups G2. G2 structures are those that have high levels of identity and were chosen for the docking molecular process [17].

### 2.4. Identification of Targets for Vaccines

The proteins predicted as secreted and present in the plasma membrane of the parasite *T. cruzi* were submitted to VaxiJen to evaluate antigenicity and immunogenicity [18]. This tool is based on the transformation of cross-auto-covariance (ACC) of protein sequences into uniform vectors of major amino acid properties [18]. Thus, ACC transformations remove the influence of sequence length. Antigenicity and immunogenicity are not simple linear properties, and the ACC physicochemical properties process adequately reflects the discrimination between antigen and nonantigen [18]. All proteins that indicated antigenicity above the cutoff (>0.7) were considered possible vaccine targets. The molecules identified were also evaluated for their similarities to proteins of other trypanosomatids to prospect targets that could be used as diagnostic tools.

### 2.5. Identification of Targets for Diagnosis

The proteins predicted as secreted and present in the plasma membrane of the parasite *T. cruzi* were also submitted to BLASTp (protein-protein blast), NCBI, to evaluate similarities to other organisms, including other trypanosomatids. This is important because the molecule could induce a great immune response and be particular to only one parasite such as the case of *T. cruzi*. All proteins that indicated antigenicity above the cutoff (>0.7) were considered possible for a diagnostic target but only one was specific for *T. cruzi*.

### 2.6. Epitope Prediction

The Immune Epitope Database and Analysis Resource (IEDB) contains a diverse catalog of information on immunogenic epitopes and immune response cells, using this information to predict and analyze epitope candidates, i.e., molecular targets of the adaptive immune response [19]. T cell epitope prediction for MHC I and II was performed through the tool PredictionMethod: IEDB recommended version 2.19, available at https://www.iedb.org/. For the epitope prediction for MHC I, the option human MHC was selected and all HLA allele references were used, and for MHC II (http://tools.iedb.org/mchii/), the following alleles were analyzed: HLA-DRB1∗03:01, HLA-DBR1∗07:01, HLA-DRB1∗15:01, HLA-DRB3∗01:01, HLA-DRB3∗02:02, HLA-DRB4∗01:01, and HLA-DRB5∗01:01. B cell epitopes were predicted using the tool found at http://tools.iedb.org/main/bcell/: linear prediction of protein sequence epitopes. Some methods are used to predict linear B cell epitopes, based on antigen sequence characteristics using amino acid scales and HMMs (*hidden Markov model*); the sequence of interest was analyzed by the BepiPred 2.0 linear epitope prediction.

### 2.7. Identification of Drug Targets

Compounds described in the literature with antiparasitic activity, whether natural, isolated from medicinal plants, or secondary metabolites, were selected and a library of ligands was created. The structures of 67 compounds were downloaded from PubChem (https://pubchem.ncbi.nlm.nih.gov/) [20] in .sdf format and converted to .PDB using the Open Babel tool (v-2.4.1) [21]. PDB format was used to assign Gasteiger atomic partial loads and convert all binders to PDBQT format using the prepare_ligand4.py script on the terminal.

The 3D structure information and drainage analysis play an important role in pathogen target prioritization and authentication [22]. The three-dimensional structure of the final drug targets identified by the MHOLline workflow (http://www.mholline.lncc.br/) was submitted to the DoGSiteScorer druggability analysis [23]. DoGSiteScorer is an automated online tool that calculates the drug's ability to interact with protein wells. For each identified cavity, the tool provides the cavity residues and a capacity score ranging from 0 to 1 [23]. Additionally, three-dimensional drug target protein structures were identified and converted to the required PDBQT format using ADT (Auto Dock Tool), MGL Tool (Version 1.5.4) [24]. For each target, a grid box in the center of the active site (comprising residues obtained from DoGSiteScorer) was created for docking analysis.

### 2.8. Molecular Docking and Virtual Screening

The ligands and proteins/receptors were submitted to the AutoDock Vina software for molecular docking analyses, using the vina_screen_local.sh script [25]. Furthermore, the best-ranked molecules were identified by the script in python topmolecule.py. The three-dimensional positions of the docking molecules were analyzed by Chimera [26], and PoseView was used for two-dimensional representations [27].

## 3. Results

**Figure 1:**
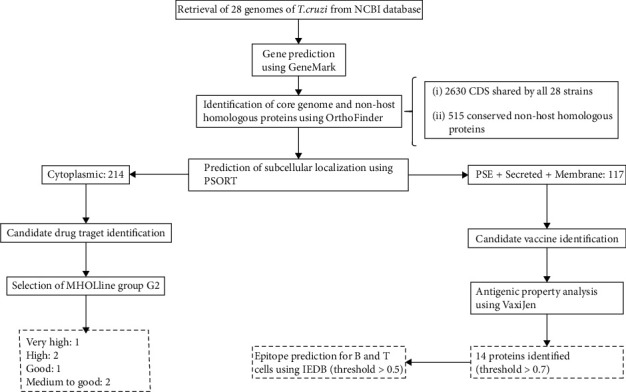
Workflow with the methods and the total number of proteins identified in each step.

The workflow of our approach, methods, and the total proteins found in each step is shown in Figure 1. We compared 28 genomes of *T. cruzi* strains (Table 1). The coding DNA sequences (CDSs) shared by all strains, known as core genome, correspond to 2630 CDSs. Among these CDSs, considering the human genome as the host genome, we found 515 conserved proteins not homologous to the host.

The subcellular localization of the proteins was predicted, in which secreted proteins (present in the endoplasmic reticulum), membrane proteins, and proteins belonging to the vesicle secretory system were selected since they are probably the most antigenic proteins and can be readily recognized by the immune system [28]. From those 515 conserved proteins not homologous to the host, 117 are secreted, membrane protein component, or proteins belonging to the vesicle secretory system (Table 2). Subsequently, these 117 proteins were submitted to VaxiJen to find proteins with a probability of MHC I and MHC II binding greater than 0.7. We found 14 proteins that are likely to be presented as antigens, from which 6 are mucin-associated surface proteins (MASPs), 6 are hypothetical proteins from different parasite strains, 1 is GP63 surface protease, and the latter was identified as putative retrotransposon hot spot protein (Table 3).

**Figure 2:**
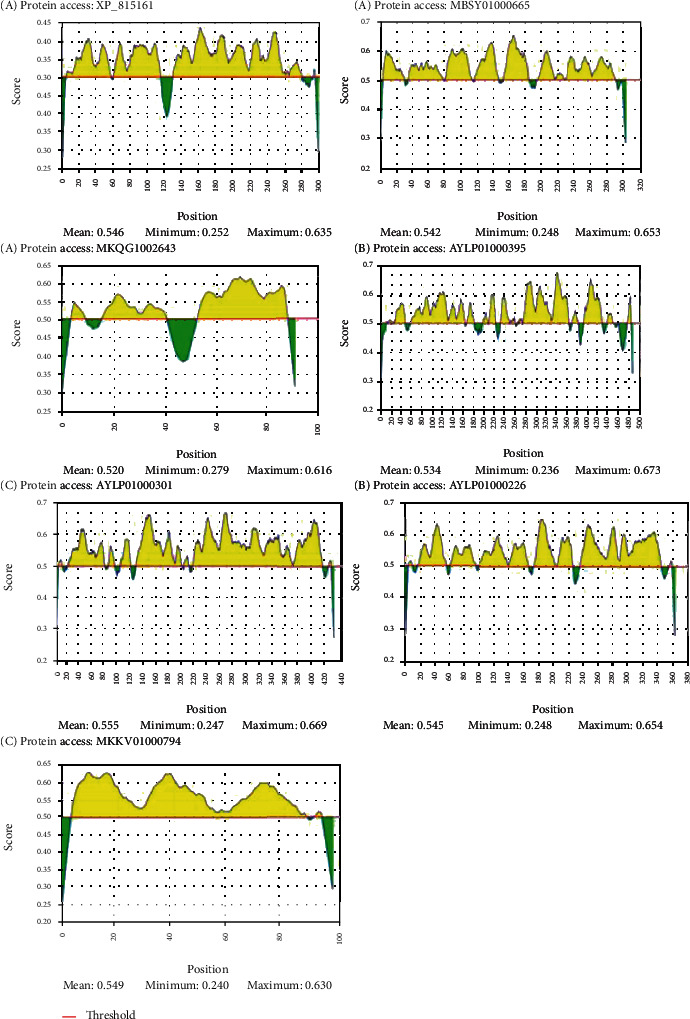
Possible epitopes that bind to B lymphocytes. (a) Epitopes present on proteins found in the endoplasmic reticulum. (b) Epitopes present on plasma membrane proteins. (c) Epitopes present in proteins of the vesicle secretory system. All amino acid sequences that exceed the cutoff line, standardized at >0.5, are considered possible yellow-labeled epitopes

The epitopes from 14 proteins identified by the VaxiJen software were predicted using the IEDB-based algorithms for both B cells and T cells (MHC I and MHC II). B cell epitope analysis was performed, and according to the previous cellular localization prediction, 100, 50, and 2 epitopes were found for those proteins present in the plasma membrane, endoplasmic reticulum, and vesicle secretory system, respectively. Graphs A, B, and C in Figure 2 demonstrate the epitopes' localization. A standard cut line greater than 0.5 was used, where the above cut epitopes are represented in yellow. The average, maximum, and minimum values are described in the legend of each graph.

HLA genes are highly polymorphic and differ among populations in both frequency and presence or absence of alleles. Thus, we used a software that classifies the epitopes according to the probability of MHC binding, in which those closer to 0 have a higher probability of binding to MHC, i.e., a greater chance of being recognized as an epitope. Due to the great diversity of alleles, the 30 best proteins classified for MHC I were selected, from which 20 are present in the plasma membrane, 9 in the endoplasmic reticulum, and only 1 in the vesicle secretory system (Table 4). For MHC II, the best 30 were also selected, being 25 present in the plasma membrane and 5 in the endoplasmic reticulum (Table 5).

**Figure 3:**
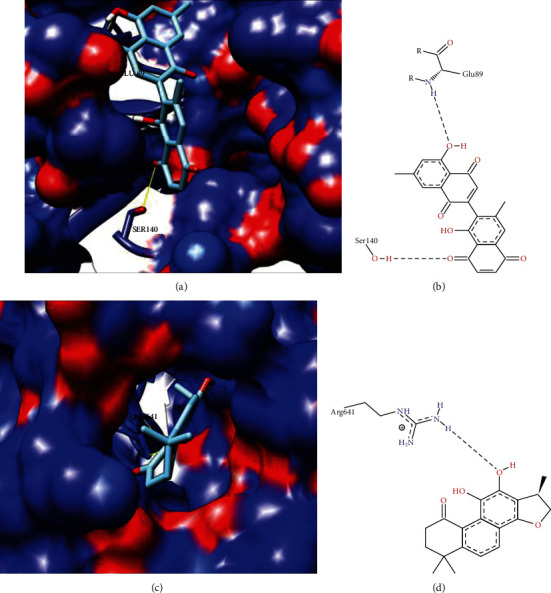
3D and 2D representation of target protein anchor analysis M18 protein RNA editing complex and hypothetical protein TCDM_03925. (a) 3D surface representation of M18 protein RNA editing complex protein with compound Diospirin. (b) 2D surface representation of M18 protein RNA editing complex protein with Diospirin compound. (c) 3D surface representation of hypothetical protein TCDM_03925 with compound (1R)-1,6,6-trimethyl-1,2,7,8-tetrahydronaphtho[1,2-g][1]benzofuran-9,10,11-trione. (d) 2D representation of the hypothetical protein TCDM_03925 with compound (1R)-1,6,6-trimethyl-1,2,8,8-tetrahydronaphto[1,2-g][1]benzofuran-9,10,11-trione.

**Figure 4:**
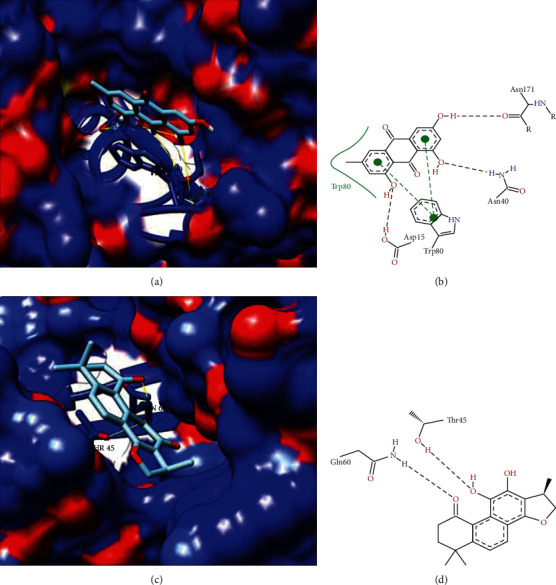
3D and 2D representation of target protein docking analysis inosine-guanine nucleoside hydrolases and mitochondrial RNA-binding protein 1. (a) 3D surface representation of inosine-guanine nucleoside hydrolases with Emodin compound. (b) 2D representation of protein inosine-guanine nucleoside hydrolases with Emodin compound. (c) 3D surface representation of protein mitochondrial RNA-binding protein 1 with (1R)-1,6,6-trimethyl-1,2,7,8-tetrahydronaphtho[1,2-g][1]benzofuran-9,10,11-trione. (d) 2D representation of RNA-binding protein mitochondrial 1 with compound (1R)-1,6,6-trimethyl-1,2,7,8-tetrahydronaphtho[1,2-g][1]benzofuran-9,10,11-trione.

**Figure 5:**
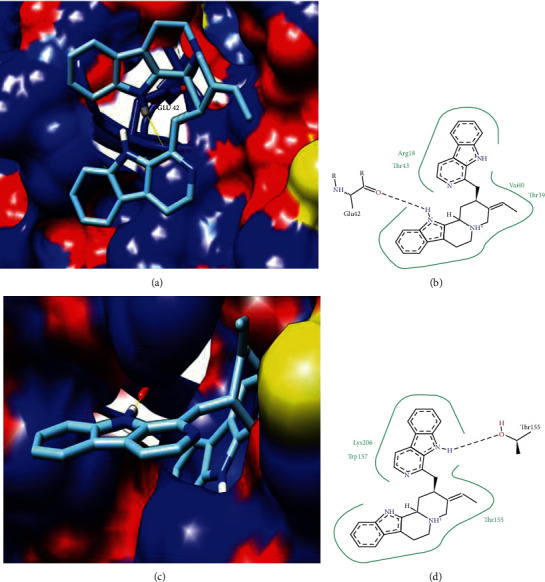
3D and 2D representation of docking analysis of the target protein calpain-like cysteine peptidase and trans-sialidase. (a) 3D surface representation of calpain-like cysteine peptidase protein with compound Usambarensine. (b) 2D representation of calpain-like cysteine peptidase protein with compound Usambarensine. (c) 3D surface representation of trans-sialidase with compound Usambarensine. (d) 2D trans-sialidase representation with the Usambarensine compound.

Currently, phytotherapics and natural plant products are frequently used in health services in both developed and developing countries and play important roles in recent drug development. They are known as a combination of chemicals that are synthesized by plants, having a moderate impact due to very low absorption by oral administration [29, 30]. The compounds selected by our group are described as medicinal plants or natural products with antiprotozoal activity against *T. cruzi*. For each target protein, all ligand compounds were used for docking analysis with drug residues in the cavity identified by the DoGSiteScorer [23] (Table 6). The best binding affinity score-based compounds generated by AutoDock Vina were analyzed for better position detection (Table 7). As a result, the predicted protein-ligand interactions for the best ligand compounds with each target that showed a significant interaction with most drug pouch residues, lower binding affinity scores, and number of hydrogen bonds are described in Table 7; moreover, we represented 3D and 2D target protein docking analysis (Figures 3–5).

## 4. Discussion

The vast repertoire of MASP sequences in the *T. cruzi* genome and the fact that they can be secreted by the parasite contribute to the ability of this protozoan to infect various host cell types and/or to participate in mechanisms of its immune evasion. MASP protein has been shown to induce the process of endocytosis in Vero cells, a process by which the parasite's trypomastigote forms actively invade host cells. Additionally, MASP peptides can elicit different antibody responses to both IgG (Immunoglobulin G) and IgM (Immunoglobulin M) and the level of antibodies to a peptide may vary after sequential passage in mice. Moreover, it has been shown that changes in the repertoire of antigenic MASP peptides may contribute to the evasion of the host immune response during the acute phase of the disease [31].

The proteomic and immunoinformatics techniques showed that several members of the MASP family, expressed in the trypomastigote form, present various MHC I and II epitopes, becoming valuable targets for vaccine development. It has been revealed that a synthetic 20-mer peptide (MASPpep) containing potential overlapping of B cells and T CD4 and T CD8 cell epitopes can induce immunity mediated by these two cell types against *T. cruzi* infection in mice. These data demonstrated that a MASPpep synthetic peptide-based vaccine can effectively control *T. cruzi* infection, prolonging survival and possibly reducing disease progression by inducing optimal immune stimulation, i.e., involving humoral and cellular responses [32]. The central region of MASP is highly variable, contributing to a vast repertoire of peptides that can interact with several receptors of different host cell types. Therefore, it is interesting to investigate whether MASP induces the immune system, especially during the acute phase of infection, when there are many circulating trypomastigotes in the human host organism [33].

Proteases are present in different protozoan parasites and appear to be important to several aspects of parasite-host interactions, regardless of their participation in pathogen nutrition [34]. Metalloproteases have been described in several parasites, but only those present in *Leishmania* spp. were completely characterized [35]. On its external surface of the plasma membrane, *Leishmania spp*. express an important 63 kDa glycosylphosphatidylinositol- (GPI-) anchored glycoprotein called gp63 or leishmanolysin, which represents more than 1% of the total cellular protein content [36, 37]. Gp63 plays several roles in parasite-host interactions and is an important virulence factor [38]. In *T. cruzi*, different metalloprotease activities have been described [39]; some of them expressed only during the metacyclogenesis phase [40, 41]. Four gp63 homologous genes have already been identified in *T. cruzi*; some of which are predominantly expressed at the mRNA level in the amastigote phase [42].

Immunocomplexes (ICs) are direct and real-time products of humoral immune responses. Among the various parasitic antigens incorporated in ICs, gp63 is relatively well known for its function [43]. Antipeptide antibodies against the C-terminal epitope, present in a subset of gp63 proteins, are recognized at all stages of the parasite and subsequently inhibit host cell trypomastigote infection [34]. *In vitro* studies also demonstrate that the presence of anti-gp63 serum has a significant inhibitory effect on *T. cruzi* infection [44].

Retrotransposon hot spot (RHS) proteins are encoded by a multigenic family present in *T. cruzi* and *Trypanosoma brucei*, but are not found in the *Leishmania* spp. genome [45]. A recent proteomic analysis was able to identify around 39 HRH isoforms that were expressed in the *T. cruzi* circulating trypomastigote form [46]. In ELISA tests, only the RHS recombinant has shown a strong serum response in patients with different clinical manifestations of Chagas disease [47]. Studies demonstrate through proteomics that *T. brucei* expresses the RSH protein [48]. However, several RHS protein sequence alignments showed that *T. cruzi* and *T. brucei* share less than 33% identity. No cross-reactivity between *T. cruzi* RHS protein in serum from patients with African sleeping sickness or leishmaniasis has been observed, thus indicating that HRH can be used as an antigen to increase the specificity of the diagnosis of Chagas disease [47]. More importantly, this protein could be tested to build a diagnostic test that could determine the clinical forms of the disease, a test that is not yet available.

Understanding antigen recognition at the molecular level opens the way to design new epitopic vaccines [49]. The identification of epitopes for both B and T cells is required to develop such vaccines since the antigenic determinants are immunodominant and capable of inducing a specific immune response [50]. Tools capable of predicting epitopes can serve as filters, i.e., they rule out regions that are probably not epitopes of additional experimental analysis [51]. This leads to the nomination of new candidates for more assertive and probably more efficient vaccines [49]. Therefore, by using reverse vaccinology, our work found possible vaccine targets that, after purification, will give rise to a prophylactic vaccine where the predicted antigens will be purified and tested *in vitro* and *in vivo* together with adjuvants, in order to generate greater efficiency.

The PDB ID: 3K81 model (*T. brucei* editosome central interaction protein in the single-domain antibody complex) crystalline structure showed identity ≥ 75% with the T M18 protein RNA editing complex target-protein I. M18 protein RNA editing complex plays an important role in the RNA editing process in the mechanism of trypanosomatids to insert and exclude posttranscriptional uridylates at multiple sites in most mitochondrial pre-mRNAs for the production of mature mRNAs [52]. RNA editing is composed of a cascade of reactions that usually begins from 3′ to 5′ in a transcript, resulting in a population of relatively edited as well as preedited and fully edited molecules for each protozoan. Mitochondrial cryptogeny was previously described participating in the development of efficient and specific chemotherapeutic targets against trypanosomatid pathogens [53, 54]. Molecular docking analysis for this protein with prepared ligand library druggability site residues showed that the Diospirin compound interacted with the active residues of GLU89 and SER140 (Table 7). Figures 3(a) and 3(b) show the three-dimensional and two-dimensional interaction, respectively, with the compound Diospirin.

The hypothetical protein TCDM_03925 showed homology to model PDB ID: 4YJ1 (*T. brucei* MRB1590-ADP crystal structure bound to poly-U RNA) with identity ≥ 50% and ≤75%. Residue ARG641 interacted with (1R)-1,6,6-trimethyl-1,2,7,8-tetrahydronaphtho[1,2-g][1]benzofuran-9,10,11-trione. The three-dimensional and two-dimensional representation with the compound (1R)-1,6,6-trimethyl-1,2,7,8-tetrahydronaphtho[1,2-g][1]benzofuran-9,10,11-trione is represented in Figures 3(c) and 3(d), respectively.

Inosine-guanine nucleoside hydrolases have homology to model ID PDB: 3FZ0 (inosine-guanosine nucleoside hydrolase- (IG-NH-) *Trypanosoma brucei*) with identity ≥ 50% and ≤75%. The nucleoside hydrolase (NH) class is common in all kingdoms except mammals, in which their absence in the host has been highly recommended as potential targets for the antitrypanosomal drug [55]. Studies have also mentioned that NH inhibitors exhibit selective inhibition of isoenzyme for IAG-NH and IG-NH due to variation in the active site characteristics, and inhibition of only one NH is not sufficient to impair the purine salvage pathway in parasites [56, 57]. Comparing the DoGSiteScorer active site identification analysis (Table 6) and our molecular docking analysis for this protein, we found that the Emodin compound showed high interaction with ASP15, TRP60, and ASN40 (Table 7) residues with good scores linked to our active site identification analysis. Figures 4(a) and 4(b) show the three-dimensional and two-dimensional interaction, respectively, with the compound Diospirin.

Mitochondrial RNA-binding protein 1 showed homology to model ID PDB: 2GIA (*T. brucei* MRP1/MRP2 crystal structures) with identity ≥ 50%. RNA-binding proteins are essential and play an important role in posttranscriptional gene regulation, coordinating the processing, storage, and control of cellular RNAs, which ultimately influences the expression of each gene in the cell [58]. Trypanosomatids have been considered for the identification of new biological mechanisms, such as RNA trans-splicing, mitochondrial RNA editing, and antigenic variation [59]. RBPs cannot be tested as drug targets because of their lack of enzymatic activity. In this sense, it is speculated that the enzymes (kinases, phosphatases, SUMO E3 ligases, and methyltransferases) responsible for these posttranslational modifications could be good candidates for new drugs [59]. Residues of the mitochondrial RNA-binding protein 1 active site, based on a DoGSiteScorer comparison active site identification analysis (Table 6) and our molecular docking analysis, found that compound (1R)-1,6,6-trimethyl-1,2,7,8-tetrahydronaphtho[1,2-g] [1]benzofuran-9,10,11-trione showed a high interaction with relapses THR45 and GLN60. Figures 4(c) and 4(d) show the 3D and 2D representation, respectively, with compound (1R)-1,6,6-trimethyl-1,2,7,8-tetrahydronaphtho[1,2-g] [1]benzofuran-9,10,11-trione.

Calpain-like cysteine peptidase showed homology to PDB model ID: 2FE0 (SMP-1 small myristoylated protein from *Leishmania major*) with identity ≥ 25% and <35%. Calpains are calcium-dependent heterodimeric cysteine peptidases that have been widely studied in mammals and exist in two major isoforms, *μ*-calpain (calpain 1) and m-calpain (calpain 2), which require micromolar concentrations and millimolar Ca2+, respectively, for its activation. These proteins are composed of a large subunit (divided into four subunits) of approximately 80 kDa and a small subunit of 28 kDa [60]. Proteases are enzymes that break the peptide bonds and are essential for numerous biological activities: peptide digestion, activation of other enzymes, immune system modulation, cell cycle participation, differentiation, and autophagy [61]. Given that the calpain protein family plays an important role in a distinct range of human disease and biological processes, it has a crucial therapeutic potential, and much has been done to develop or identify selective calpain inhibitors [62]. These proteins in trypanosomatids can turn them candidates for drug targets. In our molecular docking analysis for this protein, we found that the Usambarensine compound showed a high interaction and binding energy with the GLU42 residue (Table 6). Figures 5(a) and 5(b) show the three-dimensional and two-dimensional representation, respectively, with the compound Usambarensine.

Trans-sialidase (TS) had homology to the PDB model ID: 1WCS (*Trypanosoma rangeli* sialidase mutant exhibiting trans-sialidase activity) with identity ≥ 25% and <35%. *T. cruzi* trans-sialidase plays a key role in immunopathological events. The trans-sialidase enzyme catalyzes the displacement of glycoconjugate sialic acid molecules from the host to receptor molecules on the parasite surface. The activity of TS causes several biological effects that lead to the subversion of the host immune system, favoring both parasite survival and the establishment of chronic infection (Nardy et al., 2016). Trans-sialidase protein has been reported as a drug target against Chagas disease (Miller III; Roitberg, 2013). In our molecular docking analysis for this protein, we found that the Usambarensine compound showed notable interaction and binding energy with the THR155 residue (Table 6). Figures 5(c) and 5(d) show the three-dimensional and two-dimensional interaction, respectively, with the Usambarensine compound.

Trypanosomatids, *T. brucei* spp., *T. cruzi*, and *Leishmania spp*. cause disease in humans and animals being potentially fatal. Unfortunately, there are no effective vaccines against these parasites, and current drug treatments are highly toxic, have a low tolerance, and require long patient compliance [59]. While current drug treatments may be effective during the acute stage of infection, newer, safer, and more effective treatments against these neglected diseases are needed. Due to the toxicity and efficacy of available antiprotozoal drugs and the emergence of drug resistance, new trypanosomatid target discovery and new bioactive compounds are of utmost importance. Here, we performed subtractive genomics for drug target identification and molecular docking analysis with 6 identified drug targets. Interestingly, some of the identified targets are already reported as drug targets for Chagas disease. We prepared the binder library from a robust literature search and performed ligand-based docking. In our anchor analysis, we identified compounds such as Diospirin, Emodin, and Usambarensine showing high binding affinity with the number of targets identified.

The compound Diospirin is a plant product with a significant inhibitory effect on *Leishmania donovani* promastigote growth. This compound inhibits the catalytic activity of parasitic DNA topoisomerase I [63]. Emodin is a natural trihydroxyanthraquinone and is obtained from the roots and bark of numerous plants (particularly rhubarb and hawthorn), being an active ingredient in several Chinese herbs. It has a role as a tyrosine kinase inhibitor, an antineoplastic agent, a laxative, and a plant metabolite [64]. Usambarensine is a plant product isolated from the roots of *Strychnos usambarensis* in Central Africa. This compound exhibits strong antimalarial and cytotoxic effect activities. Its toxicity to B16 melanoma cells has been described [65].

## 5. Conclusions

Due to the absence of diagnostic tests that can determine a clinical form of the disease, it is necessary to develop a vaccine for the prevention and new drugs for the treatment of Chagas disease. Here, we apply the reverse vaccinology approach and identify 14 vaccine candidate proteins; these can also be used as a target for the diagnosis of clinical forms of the disease since it is specific for the *T. cruzi* parasite. We have also identified potential targets for already available drugs and natural products through molecular docking. We emphasize that both approaches are important but require a lot of time which can be further validated through *in vitro* and *in vivo* experiments.

## Figures and Tables

**Table 1:** General information about the 28 *T. cruzi* strains used in this work.

DTU	Strain	Protein number^1^	Size	GC (%)	Assembly
I	G	12544	25, 2	47, 4	GCA_003719455.1
I	Dm28c	29158	27, 3	50, 6	GCA_000496795.1
I	Colombiana	18033	30, 9	50, 8	GCA_003594625.1
I	B7	19174	34, 2	50, 9	GCA_000300495.1
I	Sylvio X10/1	22851	38, 6	51, 2	GCA_000188675.2
I	JR cl. 4	23024	41, 5	51, 2	GCA_000331405.1
I	Dm28c	15105	51	51, 6	GCA_002219105.2
I	Dm28c	30416	53, 3	51, 6	GCA_003177105.1
II	S44a	8043	17, 2	45	GCA_003594705.1
II	S154a	9007	19, 3	—	GCA_003594715.1
II	Ycl6	12514	25, 8	46, 6	GCA_003594465.1
II	Ycl2	12647	25, 9	46, 6	GCA_003594485.1
II	Ycl4	12734	26, 1	46, 6	GCA_003594405.1
II	S92a	12924	27, 1	46, 4	GCA_003594445.1
II	S162a	12953	27, 3	45, 3	GCA_003594605.1
II	S15	13140	27, 5	46, 2	GCA_003594585.1
II	S23b	13073	28, 1	45, 2	GCA_003594425.1
II	S11	13264	28, 5	45, 1	GCA_003594385.1
II	Y	21829	30	50, 6	GCA_003594645.1
II	Esmeraldo cl. 3	19931	38, 1	50, 6	GCA_000327425.1
II	Y	21829	39	—	GCA_002749425.1
III	231	19054	35, 4	48, 6	GCA_900252365.1
V	Arequipa	12912	19, 1	—	GCA_003594685.1
V	Bug2148	31880	55, 2	51, 3	GCA_002749415.1
VI	CL	28855	65	39, 8	GCA_003719155.1
VI	Tula cl2	44849	83, 5	51, 1	GCA_000365225.1
VI	TCC	51888	87, 1	51, 7	GCA_003177095.1
VI	CL Brener	51521	89, 9	51, 7	GCA_000209065.1

^1^GeneMarkPrediction.

**Table 2:** Subcellular localization of *T. cruzi* proteins by PSORT.

Localization	Number of proteins
Cytoplasmic	214
Nuclear	183
Endoplasmic reticulum	78
Plasma membrane	36
Vesicle secretory system	3
Vacuolar	1

**Table 3:** Potential vaccine candidates against *T. cruzi*.

Product-protein	Protein access	Subcellular location	VaxiJen (threshold > 0.7)	*E* value
MASP *T. cruzi* Dm28c	AYLP01000395	Plasma membrane	0.9138	0.0
MASP *T. cruzi* Dm28c	AYLP01000301	Plasma membrane	0.8387	0.0
MASP *T. cruzi* Dm28c	AYLP01000226	Plasma membrane	0.8157	0.0
Hypothetical protein *T. cruzi* CL Brener	XP_815161	Endoplasmic reticulum	0.7957	5*E* − 119
Hypothetical protein TcG_12168	MKKV01000794	Vesicle secretory system	0.7869	4*E* − 19
Surface protease GP63	MKQG01002643	Endoplasmic reticulum	0.7859	2*E* − 16
Hypothetical protein TCSYLVIO_002493	ADWP02012584	Plasma membrane	0.7623	3*E* − 153
MASP *T. cruzi*	MBSY01000665	Endoplasmic reticulum	0.7565	5*E* − 67
Hypothetical protein BCY84_22404	MBSY01000777	Endoplasmic reticulum	0.7431	0.0
Hypothetical protein C4B63_41g197	PRFA01000041	Plasma membrane	0.7429	0.0
MASP *T. cruzi*	MBSY01000212	Endoplasmic reticulum	0.7410	3*E* − 94
Putative retrotransposon hot spot protein	PRFA01000105	Endoplasmic reticulum	0.7402	5*E* − 40
MASP *T. cruzi* Dm28c	AYLP01000565	Plasma membrane	0.7194	0.0
Hypothetical protein TCSYLVIO_000872	ADWP02002940	Plasma membrane	0.7037	0.0

**Table 4:** Prediction of the MHC I binding.

Allele	Protein access	Size (start-end)	Peptide	Rank
HLA-A∗11:01	PRFA01000041	914-922	SAMDSMILK	0.06
HLA-A∗11:01	ADWP02002940	82-90	TTYYFAVYK	0.06
HLA-A∗33:01	ADWP02012584	146-154	DLLLYRWFR	0.06
HLA-A∗11:01	MBSY01000777	67-76	AVYDPNYLPK	0.06
HLA-B∗44:03	ADWP02002940	93-101	GEYLLIISW	0.06
HLA-A∗02:06	PRFA01000041	760-768	FVWDYFTTL	0.06
HLA-A∗02:01	MBSY01000777	219-228	FLLLFMPMFV	0.07
HLA-A∗68:01	PRFA01000041	1142-1151	SVISVITQYR	0.07
HLA-B∗53:01	MBSY01000212	705-714	LPLLLLLGLW	0.07
HLA-B∗57:01	MBSY01000665	273-282	RSTRCGFYCW	0.09
HLA-B∗15:01	ADWP02002940	113-121	FMFPDTVAF	0.1
HLA-B∗07:02	MBSY01000212	94-102	LPAKNAGAM	0.1
HLA-B∗07:02	ADWP02002940	159-167	SPRFLWIAV	0.1
HLA-B∗35:01	PRFA01000041	663-671	FPLPSSVAF	0.1
HLA-A∗68:02	PRFA01000041	726-734	MTSFFAEQV	0.1
HLA-A∗32:01	MBSY01000777	146-154	ITMFLFYAL	0.1
HLA-B∗58:01	MKKV01000794	64-72	MAAGMVILW	0.1
HLA-A∗30:01	PRFA01000041	502-510	KSRNPPLFA	0.1
HLA-A∗68:01	ADWP02002940	82-90	TTYYFAVYK	0.11
HLA-B∗44:02	PRFA01000041	487-495	AENMFMSLF	0.11
HLA-B∗44:02	ADWP02002940	93-101	GEYLLIISW	0.11
HLA-A∗26:01	PRFA01000041	1142-1150	SVISVITQY	0.11
HLA-A∗23:01	ADWP02012584	149-158	LYRWFRWYHF	0.11
HLA-A∗23:01	PRFA01000041	1112-1121	LYIAVAILSF	0.11
HLA-A∗23:01	ADWP02002940	77-86	LFMIATTYYF	0.11
HLA-B∗44:03	MBSY01000777	201-210	RESVQILWWF	0.11
HLA-B∗44:03	PRFA01000041	686-695	REFIIALKGY	0.11
HLA-A∗68:02	ADWP02002940	266-275	TTSFGVVFAV	0.11
HLA-A∗33:01	PRFA01000105	48-56	WMSLLLWLR	0.11
HLA-A∗68:01	MBSY01000665	78-86	EVAYVAAQR	0.11

**Table 5:** Prediction of the MHC II binding.

Allele	Protein access	Size (start-end)	Peptide	Rank
HLA-DRB5∗01:01	PRFA01000041	487-501	AENMFMSLFSGAKHE	0.01
HLA-DRB5∗01:01	PRFA01000041	488-502	ENMFMSLFSGAKHEK	0.01
HLA-DRB5∗01:01	PRFA01000041	489-503	NMFMSLFSGAKHEKS	0.01
HLA-DRB3∗01:01	ADWP02002940	72-86	VLTCSLFMIATTYYF	0.01
HLA-DRB3∗01:01	ADWP02002940	73-87	LTCSLFMIATTYYFA	0.01
HLA-DRB3∗01:01	ADWP02002940	74-88	TCSLFMIATTYYFAV	0.01
HLA-DRB3∗01:01	ADWP02002940	75-89	CSLFMIATTYYFAVY	0.01
HLA-DRB3∗01:01	ADWP02002940	76-90	SLFMIATTYYFAVYK	0.01
HLA-DRB3∗01:01	ADWP02002940	77-91	LFMIATTYYFAVYKQ	0.01
HLA-DRB3∗01:01	ADWP02002940	78-92	FMIATTYYFAVYKQC	0.01
HLA-DRB3∗01:01	MBSY01000777	139-153	SCAGFLFITMFLFYA	0.02
HLA-DRB3∗01:01	MBSY01000777	140-154	CAGFLFITMFLFYAL	0.02
HLA-DRB3∗01:01	MBSY01000777	141-155	AGFLFITMFLFYALS	0.02
HLA-DRB3∗01:01	MBSY01000777	217-231	PPFLLLFMPMFVAAM	0.02
HLA-DRB3∗01:01	PRFA01000041	1518-1532	ELEGSMIDLDAEVSI	0.02
HLA-DRB3∗01:01	PRFA01000041	1519-1533	LEGSMIDLDAEVSIP	0.02
HLA-DRB3∗01:01	PRFA01000041	1520-1534	EGSMIDLDAEVSIPQ	0.02
HLA-DRB3∗01:01	PRFA01000041	1521-1535	GSMIDLDAEVSIPQQ	0.02
HLA-DRB3∗01:01	PRFA01000041	1522-1536	SMIDLDAEVSIPQQK	0.02
HLA-DRB5∗01:01	PRFA01000041	486-500	HAENMFMSLFSGAKH	0.02
HLA-DRB5∗01:01	PRFA01000041	490-504	MFMSLFSGAKHEKSR	0.02
HLA-DRB5∗01:01	PRFA01000041	491-505	FMSLFSGAKHEKSRN	0.02
HLA-DRB1∗07:01	MBSY01000212	299-313	DCWVKEYVTASATMI	0.03
HLA-DRB3∗01:01	PRFA01000041	600-614	STALHAIPWDQRAFI	0.03
HLA-DRB3∗01:01	PRFA01000041	601-615	TALHAIPWDQRAFIP	0.03
HLA-DRB3∗01:01	PRFA01000041	602-616	ALHAIPWDQRAFIPI	0.03
HLA-DRB3∗01:01	PRFA01000041	603-617	LHAIPWDQRAFIPIS	0.03
HLA-DRB3∗01:01	PRFA01000041	604-618	HAIPWDQRAFIPISG	0.03
HLA-DRB3∗01:01	PRFA01000041	1108-1122	RRLILYIAVAILSFL	0.03
HLA-DRB1∗15:01	ADWP02002940	272-286	VFAVMLFGSIFVTLL	0.03

**Table 6:** Identification of the druggability cavities obtained by DoGSiteScorer.

Protein	Volume (angstrom^3^)	Surface area (angstrom^2^)	Drug score	Residues
RNA editing complex protein M18	1494.7	2197.05	0.81	VAL28, GLY29, VAL30, VAL31, HIS32, ASP33, ILE34, GLN35, THR45, GLN46, PHE47, THR48, THR50, THR51, THR52, LYS67, HIS69, ILE72, CYS74, PHE79, VAL83, LYS84, GLN85, LYS86, VAL87, LYS88, GLU89, GLY90, ASN91, VAL92, VAL93, VAL95, ASN9, VAL120, GLN126, VAL127, VAL129, HIS131, GLY132, ASP133, ARG134, ARG135, ASN136, THR137, PRO138, VAL139, SER140, VAL141, ASN142, PRO143, THR144, ALA145, GLU146
Hypothetical protein TCDM_03925	1131.67	1451.37	0.81	GLU235, ARG238, GLY239, GLU240, LEU241, ARG242, GLY245, CYS246, VAL247, ALA248, HIS300, THR337, VAL338, ASP339, PRO340, THR341, ALA342, VAL343, GLU394, LEU395, GLY396, SER397, ARG398, LEU399, GLY444, SER446, PRO503, MET504, GLN505, ARG506, LEU579, GLY582, LYS583, LEU585, LYS586, LEU587, LEU588, TYR589, SER590, PRO591, GLU596, PRO597, ARG598, ASN599, VAL600, PHE602, TYR603, SER604, GLU605, ALA607, ALA608, ILE610, GLU611, ALA639, ARG640, ARG641, PHE642, ALA659
Inosine-guanine nucleoside hydrolases	794.5	907.63	0.81	ASP11, GLY13, GLY14, ASP15, ASP16, ASN40, VAL41, THR77, VAL78, GLN79, TRP80, GLY81, GLY82, PHE83, GLY84, ARG85, ASP86, GLY87, LEU131, GLY132, PRO133, MET162, ASN171, SER172, GLU177, PHE178, ASN179, TRP205, TRP215, PHE247, LEU250, THR254, ASP273, THR275, CYS276, VAL277, ILE278, PRO279, ASP280
Mitochondrial RNA-binding protein 1	858.62	1230.81	0.82	ILE34, HIS35, ASP36, ARG38, PRO41, ALA42, LEU43, GLY44, THR45, MET46, THR47, GLN60, TYR61, PRO62, GLN63, LEU64, GLY65, ASP79, ASP81, ARG82, ILE84, ILE131, HIS132, ARG133, VAL134, ALA135, SER136, LYS138, GLU140, ASP141, TRP142, SER143, VAL144, ASN145, PHE146, ASP147, LYS148, PHE150
Calpain-like cysteine peptidase	213.06	409.93	0.27	SER6, SER7, THR8, SER9, GLU32, ILE34, GLY35, SER38, GLU42, THR43, GLY44, GLU45
Trans-sialidase	431.81	805.62	0.72	THR22, SER99, GLY100, GLY101, ALA102, GLY103, VAL104, PHE113, PRO114, TYR151, PRO152, ARG153, VAL154, THR155, LEU162, MET163, SER164, VAL165, ASP166, ARG172, VAL173, LEU190, TRP194, LYS206

**Table 7:** Molecular docking studies of all 6 drug target proteins.

Compound name	Compound PubChem ID	AutoDock Vina binding affinity	No. of H-bond/interacted residues
RNA editing complex protein M18			
Diospirin	CID308140	-7.5	2/GLU89, SER140
Hypothetical protein TCDM_03925			
(1R)-1,6,6-Trimethyl-1,2,7,8-tetrahydronaphtho[1,2-g][1]benzofuran-9,10,11-trione	CID9995530	-7.9	2/ARG641
Inosine-guanine nucleoside hydrolases			
Emodin	CID3220	-9.6	5/ASP15, TRP60, ASN40
Mitochondrial RNA-binding protein 1			
(1R)-1,6,6-Trimethyl-1,2,7,8-tetrahydronaphtho[1,2-g][1]benzofuran-9,10,11-trione	CID9995530	-9.1	4/THR45, GLN60
Calpain-like cysteine peptidase			
Usambarensine	CID5281413	-7.3	1/GLU42
Trans-sialidase			
Usambarensine	CID5281413	-9.4	1/THR155

## Data Availability

All data generated or analyzed during this study are included in this published article. Additional information about the data is available from the corresponding author on reasonable request.

## References

[1] WHO (2017). Chagas disease (American trypanosomiasis). https://www.who.int/en/news-room/fact-sheets/detail/chagas-disease-(american-trypanosomiasis.

[2] Bogliolo A. R., Lauria-Pires L., Gibson W. C. (1996). Polymorphisms in *Trypanosoma cruzi*: evidence of genetic recombination. *Acta Tropica*.

[3] Albareda M. C., Laucella S. A., Alvarez M. G. (2006). *Trypanosoma cruzi* modulates the profile of memory CD8^+^ T cells in chronic Chagas’ disease patients. *International Immunology*.

[4] Dutra W. O., Martins‐filho O. A., Cançado J. R. (1996). Chagasic patients lack CD28 expression on many of their circulating T lymphocytes. *Scandinavian Journal of Immunology*.

[5] Boscardin S. B., Torrecilhas A. C. T., Manarin R. (2010). Chagas’ disease: an update on immune mechanisms and therapeutic strategies. *Journal of Cellular and Molecular Medicine*.

[6] Dumonteil E. (2009). Vaccine development against *Trypanosoma cruzi* and *Leishmania* species in the post-genomic era. *Infection, Genetics and Evolution*.

[7] Teh-poot C., Dumonteil E. (2019). Mining Trypanosoma cruzi Genome Sequences for Antigen Discovery and Vaccine Development. *T. cruzi Infection: Methods and Protocols*.

[8] Basombrio M. A., Segura M. A., Gomez L., Mora M. C. (1993). Field trial of vaccination against American trypanosomiasis (Chagas’ disease) in dogs. *The American Journal of Tropical Medicine and Hygiene*.

[9] Basombrio M. A., Gomez L., Padilla A. M., Ciaccio M., Nozaki T., Cross G. A. M. (2002). Targeted deletion of the GP72 gene decreases the infectivity of *Trypanosoma cruzi* for mice and insect vectors. *The Journal of Parasitology*.

[10] Brandan C. P., Basombrío M. Á. (2012). Genetically attenuated *Trypanosoma cruzi* parasites as a potential vaccination tool. *Bioengineered*.

[11] Soares S. C., Trost E., Ramos R. T. J. (2013). Genome sequence of *Corynebacterium pseudotuberculosis* biovar *equi* strain 258 and prediction of antigenic targets to improve biotechnological vaccine production. *Journal of Biotechnology*.

[12] Jackie B., Sagar S., Alamgir H. (2018). in silico Molecular Docking and ADME/T Analysis of Some Selected Isolated Compounds of Phyllanthus emblica against Type 2 Diabetics. *American Journal of Ethnomedicine*.

[13] EmmsSteven Kelly D. M. (2015). OrthoFinder: solving fundamental biases in whole genome comparisons dramatically improves orthogroup inference accuracy. *Genome Biology*.

[14] DJ M. G. (1985). On the predictive recognition of signal peptide sequences. *Virus Research*.

[15] von Heijne G. (1986). A new method for predicting signal sequence cleavage sites. *Nucleic Acids Research*.

[16] (2004). Nakai K. https://psort.hgc.jp/helpwww2.html.

[17] Capriles P. V. S. Z., Guimarães A. C. R., Otto T. D., Miranda A. B., Dardenne L. E., Degrave W. M. (2010). Structural modelling and comparative analysis of homologous, analogous and specific proteins from *Trypanosoma cruzi* versus *Homo sapiens*: putative drug targets for Chagas’ disease treatment. *BMC Genomics*.

[18] Doytchinova I. A., Flower D. R. (2008). Bioinformatic approach for identifying parasite and fungal candidate subunit vaccines. *The Open Vaccine Journal*.

[19] Vita R., Overton J. A., Greenbaum J. A. (2015). The immune epitope database (IEDB) 3.0. *Nucleic Acids Research*.

[20] Kim S., Chen J., Cheng T. (2019). PubChem 2019 update: improved access to chemical data. *Nucleic Acids Research*.

[21] O’Boyle N. M., Banck M., James C. A., Morley C., Vandermeersch T. (2011). Open Babel: an open chemical toolbox. *Journal of Cheminformatics*.

[22] Jamal S. B., Hassan S. S., Tiwari S. (2017). An integrative *in-silico* approach for therapeutic target identification in the human pathogen *Corynebacterium diphtheriae*. *PLoS One*.

[23] Volkamer A., Kuhn D., Rippmann F., Rarey M. (2012). Dogsitescorer: a web server for automatic binding site prediction, analysis and druggability assessment. *Bioinformatics*.

[24] Morris G. M., Huey R., Lindstrom W. (2009). AutoDock4 and AutoDockTools4: Automated docking with selective receptor flexibility. *Journal of Computational Chemistry*.

[25] Oleg T., Olson A. J. (2010). AutoDock Vina: improving the speed and accuracy of docking with a new scoring function, efficient optimization, and multithreading. *Journal of Computational Chemistry*.

[26] Pettersen E. F., Goddard T. D., Huang C. C. (2004). UCSF Chimera?A visualization system for exploratory research and analysis. *Journal of Computational Chemistry*.

[27] Stierand K., Maaß P. C., Rarey M. (2006). Molecular complexes at a glance: automated generation of two-dimensional complex diagrams. *Bioinformatics*.

[28] Mora M., Veggi D., Santini L., Pizza M., Rappuoli R. (2003). Reverse vaccinology. *Drug Discovery Today*.

[29] Kumar Srivastava A. (2018). Significance of medicinal plants in human life. *Synthesis of Medicinal Agents from Plants*.

[30] Veeresham C. (2012). Natural products derived from plants as a source of drugs. *Journal of Advanced Pharmaceutical Technology and Research*.

[31] dos Santos S. L., Freitas L. M., Lobo F. P. (2012). The MASP family of *Trypanosoma cruzi*: changes in gene expression and antigenic profile during the acute phase of experimental infection. *PLoS Neglected Tropical Diseases*.

[32] Serna C., Lara J. A., Rodrigues S. P., Marques A. F., Almeida I. C., Maldonado R. A. (2014). A synthetic peptide from *Trypanosoma cruzi* mucin-like associated surface protein as candidate for a vaccine against Chagas disease. *Vaccine*.

[33] Bartholomeu D. C., Cerqueira G. C., Leao A. C. A. (2009). Genomic organization and expression profile of the mucin-associated surface protein (masp) family of the human pathogen *Trypanosoma cruzi*. *Nucleic Acids Research*.

[34] Cuevas I. C., Cazzulo J. J., Sánchez D. O. (2003). gp63 homologues in *Trypanosoma cruzi*: surface antigens with metalloprotease activity and a possible role in host cell infection. *Infection and Immunity*.

[35] Bouvier J., Schneider P., Etges R. (1995). Leishmanolysin: Surface metalloproteinase of Leishmania. *Proteolytic Enzymes: Aspartic and Metallo Peptidases*.

[36] Etges R., Bouvier J., Bordier C. (1986). The major surface protein of *Leishmania* promastigotes is anchored in the membrane by a myristic acid-labeled phospholipid. *The EMBO Journal*.

[37] Bouvier J., Etges R. J., Bordier C. (1985). Identification and purification of membrane and soluble forms of the major surface protein of *Leishmania* promastigotes. *The Journal of Biological Chemistry*.

[38] Joshi P. B., Kelly B. L., Kamhawi S., Sacks D. L., McMaster W. R. (2002). Targeted gene deletion in *Leishmania major* identifies leishmanolysin (GP63) as a virulence factor. *Molecular and Biochemical Parasitology*.

[39] Greig S., Ashall F. (1990). Electrophoretic detection of *Trypanosoma cruzi* peptides. *Mol Biochem Parasitol*.

[40] Bonaldo M. C., d'Escoffier L. N., Salles J. M., Goldenberg S. (1991). Characterization and expression of proteases during *Trypanosoma cruzi* metacyclogenesis. *Experimental Parasitology*.

[41] Lowndes C. M., Bonaldo M. C., Thomaz N., Goldenberg S. (1996). Heterogeneity of metalloprotease expression *inTrypanosoma cruzi*. *Parasitology*.

[42] Grandgenett P. M., Coughlin B. C., Kirchhoff L. V., Donelson J. E. (2000). Differential expression of GP63 genes in *Trypanosoma cruzi*. *Molecular and Biochemical Parasitology*.

[43] Ohyama K., Huy N. T., Yoshimi H. (2016). Proteomic profile of circulating immune complexes in chronic Chagas disease. *Parasite Immunol*.

[44] Kulkarni M. M., Olson C. L., Engman D. M., McGwire B. S. (2009). *Trypanosoma cruzi* GP63 proteins undergo stage-specific differential posttranslational modification and are important for host cell infection. *Infection and Immunity*.

[45] Bringaud F., Biteau N., Melville S. E. (2002). A new, expressed multigene family containing a hot spot for insertion of retroelements is associated with polymorphic subtelomeric regions of *Trypanosoma brucei*. *Eukaryotic Cell*.

[46] Brunoro G. V. F., Caminha M. A., Ferreira A. T. S. (2015). Reevaluating the *Trypanosoma cruzi* proteomic map: the shotgun description of bloodstream trypomastigotes. *Journal of Proteomics*.

[47] Bautista-López N. L., Ndao M., Camargo V. (2017). Characterization and diagnostic application of *Trypanosoma cruzi* trypomastigote excreted-secreted antigens shed in extracellular vesicles released from infected mammalian cells. *Journal of Clinical Microbiology*.

[48] Geiger A., Hirtz C., Bécue T. (2010). Exocytosis and protein secretion in *Trypanosoma*. *BMC Microbiology*.

[49] Raza S., Siddique K., Rabbani M. (2019). *In silico* analysis of four structural proteins of aphthovirus serotypes revealed significant B and T cell epitopes. *Microbial Pathogenesis*.

[50] Dermime S., Gilham D. E., Shaw D. M. (2004). Vaccine and antibody-directed T cell tumour immunotherapy. *Biochimica et Biophysica Acta (BBA) - Reviews on Cancer*.

[51] Jespersen M. C., Peters B., Nielsen M., Marcatili P. (2017). BepiPred-2.0: improving sequence-based B-cell epitope prediction using conformational epitopes. *Nucleic Acids Research*.

[52] Panigrahi A. K., Schnaufer A., Carmean N. (2001). Four related proteins of the *Trypanosoma brucei* RNA editing complex. *Molecular and Cellular Biology*.

[53] Gerasimov E. S., Gasparyan A. A., Kaurov I. (2018). Trypanosomatid mitochondrial RNA editing: dramatically complex transcript repertoires revealed with a dedicated mapping tool. *Nucleic Acids Research*.

[54] Salavati R., Moshiri H., Kala S., Shateri N. H. (2012). Inhibitors of RNA editing as potential chemotherapeutics against trypanosomatid pathogens. *International Journal for Parasitology: Drugs and Drug Resistance*.

[55] Giannese F., Berg M., van der Veken P. (2013). Structures of purine nucleosidase fromTrypanosoma bruceibound to isozyme-specific trypanocidals and a novel metalorganic inhibitor. *Acta Crystallographica Section D: Biological Crystallography*.

[56] Berg M., Kohl L., Van der Veken P. (2010). Evaluation of nucleoside hydrolase inhibitors for treatment of african trypanosomiasis. *Antimicrobial Agents and Chemotherapy*.

[57] CHH H., Fatima A., Gaurav A. (2015). In Silico Investigation of Flavonoids as Potential Trypanosomal Nucleoside Hydrolase Inhibitors. *Advances in Bioinformatics*.

[58] Antonicka H., Sasarman F., Nishimura T., Paupe V., Shoubridge E. A. (2013). The Mitochondrial RNA-Binding Protein GRSF1 Localizes to RNA Granules and Is Required for Posttranscriptional Mitochondrial Gene Expression. *Cell Metabolism*.

[59] Romaniuk M. A. (2016). Regulation of RNA binding proteins in trypanosomatid protozoan parasites. *World Journal of Biological Chemistry*.

[60] Branquinha M., Marinho F., Sangenito L. (2013). Calpains: potential targets for alternative chemotherapeutic intervention against human pathogenic trypanosomatids. *Current Medicinal Chemistry*.

[61] Siqueira-Neto J. L., Debnath A., McCall L.-I. (2018). Cysteine proteases in protozoan parasites. *PLOS Neglected Tropical Diseases*.

[62] Saez M. E., Ramirez-Lorca R., Moron F. J., Ruiz A. (2006). The therapeutic potential of the calpain family: new aspects. *Drug Discovery Today*.

[63] Ray S., Hazra B., Mittra B., Das A., Majumder H. K. (1998). Diospyrin, a bisnaphthoquinone: a novel inhibitor of type I DNA topoisomerase of *Leishmania donovani*. *Molecular Pharmacology*.

[64] Dong X., Fu J., Yin X. (2016). Emodin: a review of its pharmacology, toxicity and pharmacokinetics. *Phytotherapy Research*.

[65] Dassonneville L., Wattez N., Mahieu C. (1999). The plant alkaloid usambarensine intercalates into DNA and induces apoptosis in human HL60 leukemia cells. *Anticancer research*.

